# Exogenous insulin antibody syndrome presenting with concurrent hypoglycemia and hyperglycemia: A case report following complete pancreatectomy

**DOI:** 10.1097/MD.0000000000050281

**Published:** 2026-08-28

**Authors:** Xiao-Po Wang, Yan Wu, Jing Wu, Ying-Zhao Liu, Hui Jiang

**Affiliations:** a Department of Endocrinology, the Affiliated People’s Hospital of Jiangsu University, Zhenjiang, Jiangsu, China.

**Keywords:** exogenous insulin antibody syndrome, hyperglycemia, hypoglycemia, total pancreatectomy

## Abstract

**Rationale:**

Antibodies against therapeutic insulin cause paradoxical hypoglycemia, termed exogenous insulin antibody syndrome (EIAS). This first report of glargine-induced EIAS post-total pancreatectomy introduces mycophenolate mofetil (MMF) as a novel steroid-sparing therapy.

**Patient Concerns:**

A 75-year-old woman developed refractory nocturnal hypoglycemia with daytime hyperglycemia (HbA1c 8.6%) 3 months post-pancreatectomy during glargine therapy.

**Diagnoses:**

Hypoglycemia (glucose 2.83 mmol/L) featured undetectable C-peptide, elevated insulin (103 μU/mL), high insulin autoantibodies (74.95 coi, Reference range 0–1.0), and polyethylene glycol (PEG) precipitation-confirmed antibody-bound insulin, with a marked decline in insulin concentration relative to baseline values (88% decline).

**Interventions:**

Standard approaches (switching to aspart insulin, carbohydrate enrichment, degludec/acarbose triple regimen) failed; prednisone alleviated hypoglycemia but provoked hyperglycemia.

**Outcomes:**

Mycophenolate mofetil (MMF) eliminated hypoglycemia within 2 weeks, reducing antibodies to 35.4 coi.

**Conclusion:**

This inaugural case highlights EIAS as a cause of unexplained hypoglycemia in pancreatectomized patients. MMF warrants consideration as primary immunosuppressive therapy for EIAS.

**Lessons:**

This case highlights that EIAS is a rare but important cause of paradoxical hypoglycemia and hyperglycemia in insulin-treated patients. MMF can serve as an effective therapeutic agent.

## 1. Introduction

Autoimmune insulin syndrome (IAS) constitutes a rare etiology of hypoglycemia in insulin-untreated individuals, mediated by pathogenic anti-insulin autoantibodies.^[1,2]^ A parallel condition – exogenous insulin antibody syndrome (EIAS) – manifests in diabetic patients when antibodies develop against therapeutic insulin, resulting in fasting hypoglycemia and postprandial hyperglycemia.^[3,4]^ EIAS remains exceptionally uncommon,^[5]^ with only one documented case following insulin therapy after total pancreatectomy.^[6]^ We describe the inaugural documentation of glargine-induced EIAS in a totally pancreatectomized patient, establishing mycophenolate mofetil (MMF) as a clinically effective steroid-sparing intervention. Notably, MMF administration achieved a significantly accelerated resolution compared to conventional steroid regimens, while avoiding corticosteroid-associated hyperglycemia – a critical therapeutic advantage in insulin-deficient states.

## 2. Case report

A 75-year-old female underwent total pancreatectomy for intraductal papillary mucinous neoplasm (IPMN), resulting in absolute insulin dependence. Postoperatively, she developed decreased appetite and early satiety, manifestations consistent with pancreatic exocrine insufficiency (PEI) – a common complication after pancreatic surgery that may lead to digestive and absorptive disorders and malnutrition, thereby affecting patient recovery and quality of life.^[7]^ Glycemic control was initially maintained via continuous subcutaneous aspart infusion, transitioning subsequently to daily glargine (8 units) with stable euglycemia.Three months postoperatively, recurrent symptomatic nocturnal hypoglycemia emerged (self-monitored glucose ≤ 2.8 mmol/L). These events persisted despite halving the glargine dosage to 4 units daily. Admission evaluation excluded sulfhydryl medication exposure and preexisting hypoglycemic disorders. Comorbidities included controlled Hashimoto’s thyroiditis (levothyroxine 50 μg/day) and mild anemia (Hb 102 g/L). Hepatic, renal, and thyroid function profiles remained within normal limits. In addition, the laboratory value showed a higher glycosylated hemoglobin. When hypoglycemia occurs, C-peptide levels are extremely low, but insulin levels are inappropriately elevated. Insulin antibody levels increased. To further characterize insulin autoantibody interference, a polyethylene glycol (PEG) precipitation assay was performed. Immunoreactive insulin in the supernatant was quantified via electrochemiluminescence immunoassay (Roche Cobas e411). PEG precipitation demonstrated a > 40% reduction in insulin concentration relative to baseline (103 vs 12.8 μU/mL; 88% decline) (Table 1), confirming substantial antibody-mediated insulin sequestration.^[8]^

**Table 1:** Laboratory value.

Test term	Measured value
HbA1c(%)	8.6
blood glucose(mmol/l)	2.83
C-peptide (ng/ml)	<0.01
Insulin before PGE(uU/ml)	103
Insulin post PGE(uU/ml)	12.8
IAA (0–1.0 coi)	74.95
GAD-Ab(0–10 iu/ml)	1.23
ICA (0–1.0 coi)	0.24
IA-2A (0–10 iu/ml)	1.35
Zn-T8 (0–10 AU/ml)	2.32

HbA1c = glycosylated hemoglobin, PGE = polyethylene glycol, IAA = insulin autoantibody, GAD-Ab = glutamic acid decarboxylase antibody, ICA = islet cell antibody, IA-2A = tyrosine phosphatase antibody, ZnT8 = zinc transporter 8 antibody, coi = cutoff index.

**Figure 1.**
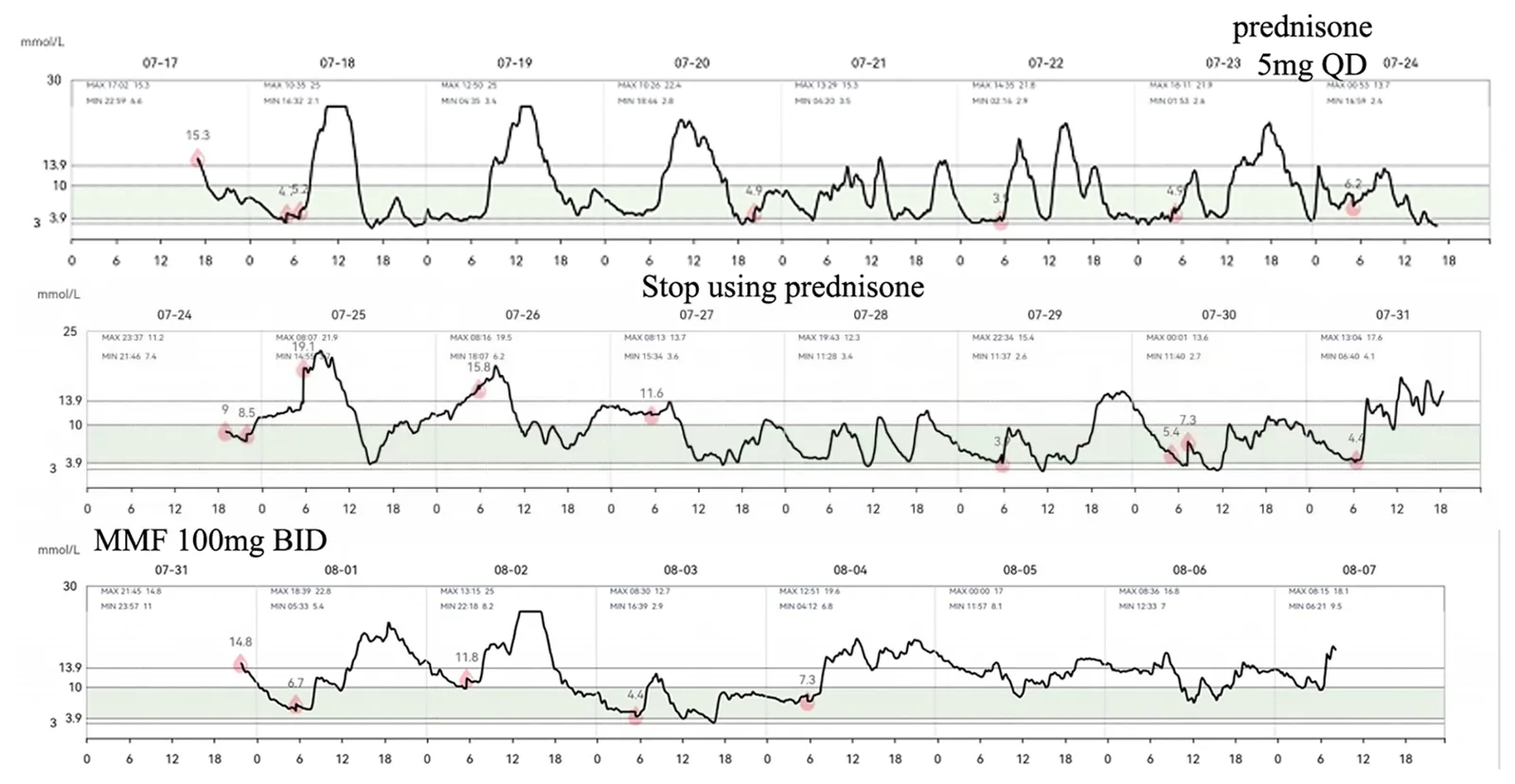
Ambulatory glucose profile of the patient following the initiation of immunosuppressive therapy. Prednisone (5 mg daily) was commenced on 23 July and discontinued after 4 days. Mycophenolate mofetil (100 mg twice daily) was initiated on 31 July.

Glargine therapy was discontinued and replaced with preprandial subcutaneous aspart insulin. Continuous glucose monitoring was initiated using a Yuwell Anytime continuous glucose monitoring system (Yuwell, a professional medical device brand from China), which recorded interstitial glucose levels at 3-minute intervals. Despite the implementation of nocturnal insulin withdrawal and bedtime nutritional supplementation, refractory nocturnal hypoglycemia (glucose ≤ 3.0 mmol/L) and persistent daytime postprandial hyperglycemia (peak glucose > 11.1 mmol/L) persisted (Fig. 1).

**Figure 2.**
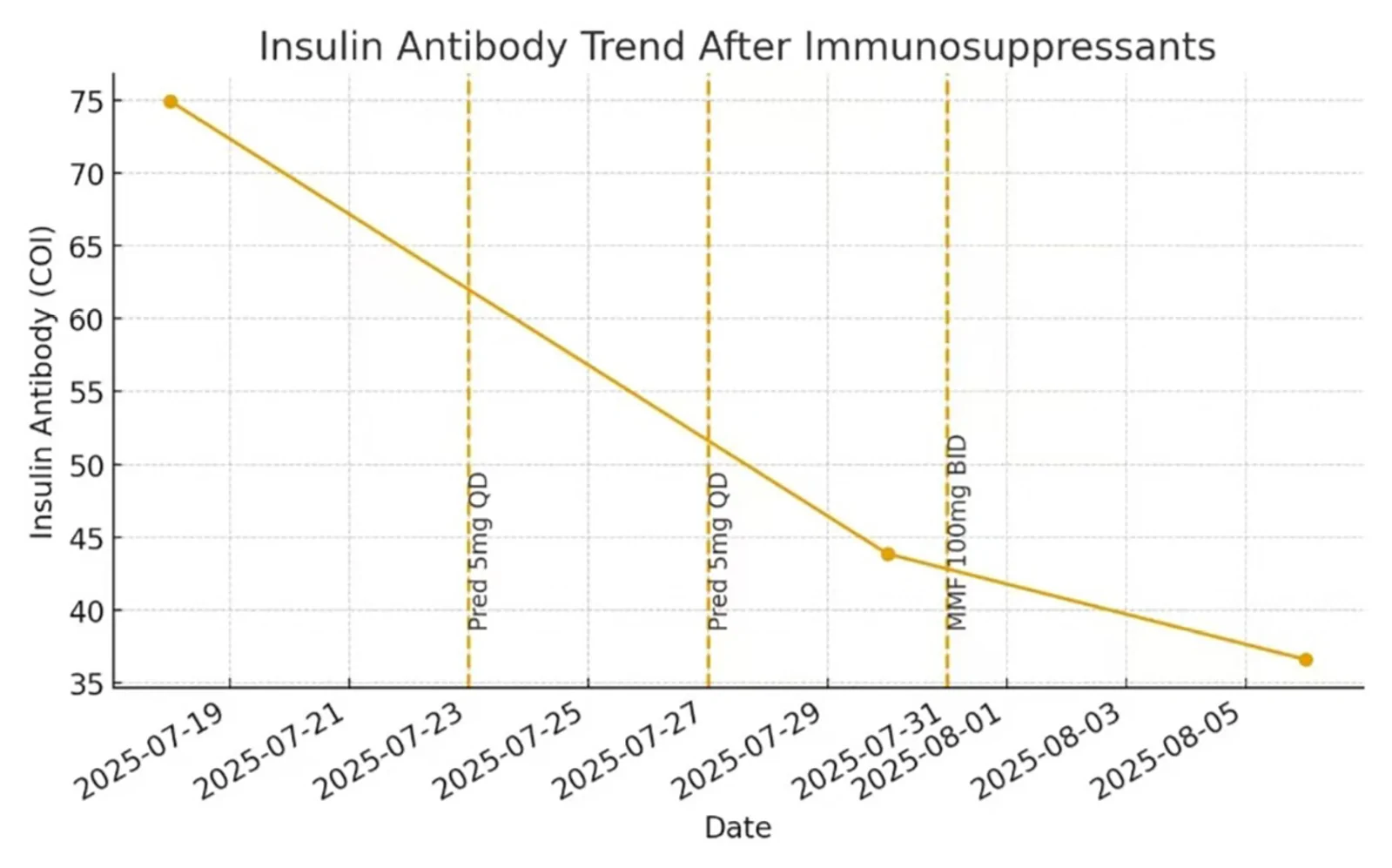
Trends in insulin antibody levels following the initiation of immunosuppressive therapy. Prednisone (5 mg daily) was administered from 23 July for 4 days. Mycophenolate mofetil (100 mg twice daily) was initiated on 31 July.

Therapeutic management was complicated by the patient’s absolute insulin dependence. A triple regimen comprising preprandial aspart insulin (3 units), nocturnal degludec (2 units), and acarbose (50 mg 3 times daily) failed to prevent hypoglycemic events, indicating an insulin-independent pathological mechanism. Subsequent low-dose prednisone (5 mg nocte) eliminated hypoglycemia but induced significant dawn hyperglycemia (15 mmol/L), highlighting the therapeutic dilemma of corticosteroid therapy. MMF led to faster resolution with reduced hypoglycemic episodes and no induction of hyperglycemia (Fig. 1). Following the substitution of prednisone with MMF, insulin antibodies exhibited a progressive decline (Fig. 2).

Given the limited treatment duration, continued monitoring is needed. During the 4-month regular outpatient follow-up after treatment, no further hypoglycemic events were documented.

## 3. Discussion

This manuscript presents the inaugural report of glargine-induced EIAS in a totally pancreatectomized patient, highlighting unique diagnostic and therapeutic challenges in absolute insulin deficiency. Crucially, it identifies MMF as an effective steroid-sparing intervention.

Diagnostic criteria for EIAS include: spontaneous hypoglycemia; elevated insulin during hypoglycemia; insulin autoantibodies linked to exogenous insulin.^[9]^ Our patient fulfilled all criteria: persistent nocturnal hypoglycemia despite aggressive insulin reduction; biochemical confirmation of discordant insulin/C-peptide levels; and markedly elevated insulin autoantibodies (IAAs). A > 40% decline in immunoreactivity post-PEG precipitation confirmed antibody-mediated insulin sequestration. This case uniquely leverages the pancreatectomized state: absent endogenous insulin and C-peptide exclude insulinoma and autoimmune hypoglycemia, providing an unconfounded model of exogenous insulin-antibody dysregulation. Coexisting hyperglycemia (HbA1c 8.6%) and recurrent hypoglycemia exemplify hallmark IAS/EIAS glycemic instability stemming from stochastic immune complex dissociation.^[1]^

EIAS pathogenesis involves high-affinity polyclonal IgG antibodies against therapeutic insulin, forming complexes that prolong insulin half-life. Spontaneous dissociation causes unregulated insulin release and hypoglycemia.^[10]^ Similar to IAS, EIAS associates with HLA susceptibility alleles (e.g., DRB1*0406/0403) and autoimmune comorbidities.^[11]^ Our patient’s Hashimoto thyroiditis suggests autoimmune predisposition may contribute to EIAS development. While animal insulins confer higher immunogenic risk, analogue-induced EIAS is increasingly recognized. Glargine’s modified pharmacokinetics and potential immunogenicity warrant further investigation.^[12]^ EIAS predominantly affects insulin-treated diabetics; this represents the first glargine-associated case post-total pancreatectomy.

EIAS management requires balancing hypoglycemia prevention against hyperglycemia risk, complicated by mandatory insulin replacement. Conventional approaches (insulin discontinuation, regimen adjustments) provided partial benefit. Persistent hypoglycemia despite optimized triple therapy (aspart/degludec/acarbose) confirmed antibody-mediated pathology, as acarbose controls postprandial hyperglycemia while degludec delivers stable basal coverage.

Immunosuppression is indicated for severe refractory IAS/EIAS. Corticosteroids suppressed hypoglycemia but provoked significant hyperglycemia, revealing a critical therapeutic dilemma in insulin-deficient states.^[13]^ MMF administration achieved an obvious reduction in IAAs, correlating with hypoglycemia resolution. By inhibiting inosine monophosphate dehydrogenase, MMF selectively suppresses lymphocyte proliferation and antibody production. Despite established roles in transplantation and autoimmunity, evidence for MMF in IAS/EIAS remains limited.^[14]^ This case illustrates that steroid-sparing properties enable modulation of immune dysregulation without triggering corticosteroid-induced hyperglycemia, thus addressing the clinical dilemma of immunosuppression in patients with insulin-dependent diabetes.

Furthermore, comprehensive post-pancreatectomy care requires nutritional support and pancreatic enzyme replacement therapy, as PEI is a frequent complication that can compromise nutritional status and long-term recovery. Personalized nutrition management after discharge is particularly important for improving nutritional status and prognosis of patients after pancreatic surgery, as emphasized in a recent study protocol by Pizzocaro E et al.^[7]^

## 4. Conclusion

This inaugural documentation of glargine-triggered EIAS post-total pancreatectomy emphasizes the imperative for EIAS evaluation in pancreatectomized patients manifesting unexplained hypoglycemia during insulin therapy. MMF demonstrates compelling therapeutic efficacy as a steroid-sparing intervention. These findings establish MMF as a primary immunosuppressive alternative for antibody-mediated hypoglycemia, particularly when glucocorticoids are contraindicated. As a single case report, robust conclusions regarding optimal therapeutic management – particularly concerning MMF long-term efficacy and safety profile in EIAS – must be validated through large-scale prospective studies with longitudinal data collection.

## Acknowledgments

The authors thank Professor Ling Li from the Affiliated Hospital of Southeast University for her guidance in the diagnosis and treatment process of this patient.

## Author contributions

**Writing – original draft:** Xiao-po Wang.

**Conceptualization:** Yan Wu.

**Project administration:** Yan Wu, Ying-Zhao Liu.

**Data curation:** Jing Wu.

**Investigation:** Jing Wu.

**Methodology:** Ying-Zhao Liu, Hui Jiang.

**Writing – review & editing:** Hui Jiang
